# Mediating effect of depression and acute stress between exposure to Israel-Gaza war media coverage and insomnia: a multinational study from five arab countries

**DOI:** 10.1186/s12889-024-18996-8

**Published:** 2024-06-04

**Authors:** Feten Fekih-Romdhane, Mai Helmy, Amthal Alhuwailah, Hanaa Ahmed Mohamed Shuwiekh, Abdallah Y. Naser, Emna Maalej, Sahar Obeid, Majda Cheour, Souheil Hallit

**Affiliations:** 1 Faculty of Medicine of Tunis, Tunis Al Manar University, Tunis, Tunisia; 2 The Tunisian Center of Early Intervention in Psychosis, Department of Psychiatry Ibn Omrane, Razi Hospital, Tunis, Tunisia; 3 Department of Psychology, Kuwait University, Kuwait, Kuwait; 4 Menoufia University, Shebin El-Kom, Egypt; 5 Psychology department, College of education, Sultan Qaboos University, Muscat, Oman; 6 Department of Psychology, Fayoum University, Faiyum, Egypt; 7 Badr University in Cairo (BUC), Cairo, Egypt; 8 Department of Applied Pharmaceutical Sciences and Clinical Pharmacy, faculty of pharmacy, Isra University, Amman, Jordan; 9 School of Arts and Sciences, Social and Education Sciences Department, Lebanese American University, Jbeil, Lebanon; 10 School of Medicine and Medical Sciences, Holy Spirit University of Kaslik, P.O. Box 446, Jounieh, Lebanon; 11 Psychology Department, College of Humanities, Effat University, Jeddah, 21478 Saudi Arabia; 12 Applied Science Research Center, Applied Science Private University, Amman, Jordan

**Keywords:** Media exposure, War media coverage, Insomnia, Depression, Acute stress, Mediation

## Abstract

**Background:**

In the context of persistent wars and conflicts worldwide, the impact of acute, excessive and constant exposure to media coverage of such events on mental health outcomes becomes a serious problem for public health, and requires therefore urgent investigation to inform an effective prevention and management response. The objective of the present study was to test the hypothesis that war-related media exposure is directly and indirectly associated with insomnia through depression and perceived stress among adults from the general population of different Arab countries.

**Methods:**

A cross-sectional study was carried-out two weeks after the beginning of Israel-Gaza war on the 7th of October 2023. An anonymous online survey and a snowball sampling method were adopted to collect data. A sample of 2635 general population adults (mean age of 23.98 ± 7.55 years, 73.1% females) took part of this study.

**Results:**

The results of the mediation analysis showed that, after adjusting over potential confounders, depression and perceived stress fully mediated the association between war media exposure and insomnia; higher war media exposure was significantly associated with higher depression (Beta = 0.13; *p* < .001) and perceived stress (Beta = 0.07; *p* < .001), whereas higher depression (Beta = 0.43; *p* < .001) and perceived stress (Beta = 0.31; *p* < .001) were significantly associated with higher insomnia. It is of note that war media exposure was not significantly and directly associated with insomnia (Beta = − 0.01; *p* = .178 and Beta = 0.02; *p* = .098 respectively).

**Conclusion:**

The present study is the first to provide evidence that more time spent viewing the horrors of war is significantly associated with insomnia. In addition, symptoms of stress and depression were present as early as two weeks following the beginning of the war, and played a significant role in mediating the association between war media coverage and insomnia. These findings suggest that timely screening for, and management of depression and stress symptoms in clinical and preventive programs might be beneficial for community adults who have been heavily and indirectly exposed to war through media, and present with insomnia.

## Introduction

Insomnia is defined as symptoms of difficulty initiating and maintaining sleep, awakening with difficulty falling back asleep with sleep that is non-restorative, and/or subjective complaints in daytime activities. Insomnia is considered a core characteristic of the human physiological neurobiological and response to trauma, and has been the subject of growing empirical interest in the field of trauma and sleep research [1]. Research on sleep problems precipitated by trauma has predominantly focused on direct exposure to traumatic events [2]. Extensive literature reported that an array of sleep disturbances can develop amongst people exposed to various sorts of traumatic events, including natural disasters (e.g., Hurricane Katrina [3], Wenchuan earthquake [4], the 2011 Japan earthquake [5]) and human-made disasters (e.g., the Vietnam war [6], the 2011 Utøya, Norway, Terror Attack [7]). There is also evidence, albeit more limited, to support that indirect exposure to trauma via mass media is associated with a range of mental health problems similar to those experienced after direct trauma exposure [8, 9], including enduring and marked patterns of sleep disruption.

Even though some previous studies documented that different types of violent media consumption is linked to increased sleep problems, including longer sleep latency, frequent night waking, shorter sleep duration and difficulty waking [10, 11], less literature has addressed this association following disasters and trauma. Bui et al. [12] found that the amount of television and Internet viewing within the two first weeks following the March 11, 2011, Japanese earth-quake and tsunami (which killed 14,000 people) significantly correlated with disruptive nocturnal behavior in general population adults from France, Canada, and the United States, suggesting that media coverage of a distant disaster can induce sleep problems. As for research related to human-made disasters, a study among 1878 adult from the French general population found that both traditional (Newspaper, Radio, TV) and social media (Youtube, Facebook, Twitter) consumption were significantly related to insomnia symptoms a month after the 2015 Bataclan terror attacks in Paris, France, with social media use having an independent additional relationship with insomnia beyond traditional media use [13].

Altogether, witnessing traumatic events through media can significantly disrupt sleep continuity and integrity in exposed individuals. Moreover, trauma-induced sleep problems appear to play a role in the induction of emotional and behavioral problems [14] and the aggravation of post-disaster difficulties [15]. Therefore, understanding how indirect exposure to information relevant to the latest Israel-Gaza war through media content may relate to sleep problems in the general public is an important topic of investigation. Some mechanisms have been advanced to explain the relationship between violent media use and sleep problems. One plausible explanation was that media content may provoke cognitive or emotional arousal, thus contributing to difficulty in falling asleep and leading to poor quality of sleep [16]. Other possible explanations could be increased depression and stress symptoms subsequent to violent media exposure, which will be the focus of this paper.

### Depression and perceived stress as mediators between war media exposure and insomnia

Beyond sleep problems, numerous other reactions to indirect or remote disaster exposure via media may also be experienced, such as depression and acute stress. A meta-analysis encompassing 20 studies mostly focusing on manmade disasters (e.g., the September 11, 2001, attacks, *n* = 9; the Israel-Palestine conflict, *n* = 5) indicated that a statistically significant positive relationship exists between mass trauma media consumption and depression [17]. There is evidence from studies performed following mass traumatic events to suggest that indirect exposure through media is sufficient to induce post-traumatic stress symptoms, and to increase community PTSD prevalence [18]. However, only a modest attention has been given to the association between acute stress reactions and contact with media following a number of collective traumas, such as the September 11 attack [19, 20], the Boston Marathon bombings [21], and the Iraq War [22]. A meta-analytic review by Pfefferbaum et al. [23] concluded to a small but statistically significant effect of viewing mass trauma media coverage on acute stress reactions.

On the other hand, both depression and acute stress were shown to lead to insomnia. In fact, although there has been a large and strong theoretical assumption and empirical evidence that insomnia is a symptom and result of depression, a more limited amount of literature is found regarding depression as a predictor of insomnia [24]. Research revealed that depression may engender disruptions in sleep patterns [25]. Depressive symptoms and depression were found to be the most consistent and largest risk factors for insomnia [26, 27], and that insomnia may develop after depression [28]. In this particular case, depression causes an increased activity of the central nervous system and a somatic arousal, which are, in turn, naturally balanced with a systemic response corresponding to insomnia to help maintain homeostasis [29]. Findings from a large multinational European study revealed that, in people with co-occurring conditions, depression symptoms appeared before onset of insomnia in 29% of cases [30]. Insomnia was also found to newly emerge in outpatients with depression who are under antidepressants [31]. Likewise, stress levels serve in impairing subsequent sleep processes [32]. There is evidence that stress experienced after adverse events is closely associated with [33], and predictor of [34] onset and maintenance of insomnia. In sum, these observations drive the theoretically-based hypothesis that both depression and perceived stress could serve as intermediate processes in the association between war-related media exposure and insomnia symptoms.

### The current study

Since October 2023, Palestinian civilians are experiencing a catastrophic situation qualified by NGOs and many international institutions (including Médecins Sans Frontières and the United Nations) as a major humanitarian crisis [35]. Over 35,000 Palestinian citizens were killed under Israel Defense Forces bombardment, a further 80,000 injured and over two million displaced [36]. Dozens of Palestinian health workers and journalists have been killed on duty, multiple residential buildings have been destroyed, families were forcibly displaced, supplies of food, water, and electricity were cut off [37]. Mass media and social media coverage of the war-related horrors has been nonstop, and have had a wide reach extending to populations in all parts of the world. This has led the global population to be constantly and repeatedly in contact with a very distressing and traumatic media content, which may have deleterious consequences on mental health, especially since evidence indicated that indirect effects of such content can be even greater in geographically-distant people than in those living in the city where the event occurred [38]. In the context of persistent wars and conflicts worldwide, the impact of acute, excessive and constant exposure to media coverage of such events on mental health outcomes becomes a serious problem for public health, and requires therefore urgent investigation to inform an effective prevention and management response. This is particularly relevant in the context of Middle East countries, where people have been experiencing pre-existing mental health vulnerabilities over the last decades. Indeed, prevalence estimates of mental health problems are reported to be high in Arab countries and the Middle East, and are predicted to increase due to ongoing conflicts and wars (for review, see [39]). All middle Eastern countries have higher levels of burden from mental illness than globally [40]. Large population-based studies in the region observed a prevalence of mental disorders ranging between 15.6% and 35.5%, with the highest rates being reported in nations with complex emergency circumstances [41]. In addition, the 12-month prevalence of mental disorders in the region was found to range from 11.0 to 40.1% [42]. More specifically, the prevalence rate of depressive disorders was found to be higher than the global average, and to have increased by 0.004% between 1990 and 2019 in the MENA region [43]. The prevalence of post-traumatic stress was found to be of 26.0-40.3% in Lebanese [44], 33.0% in Tunisian [45], 60% in Syrian [46] adults. The prevalence of insomnia across Arab countries (63.9%) was also shown to be higher compared to the rest of the world [47]. Furthermore, data on sleep in the aftermath of trauma in community adults from Arab countries are scarcer. Even though exposure to disasters is a global phenomenon, post-trauma responses were attested to differ substantially across cultures [48], and existing results on the topic from Western samples are not representative of all human and may be non-generalizable to non-western countries. Motivated by the above observations and considerations, the objective of the present study was to test the hypothesis that war-related media exposure is directly and indirectly associated with insomnia through depression and perceived stress among adults from the general population of different Arab countries.

## Methods

### Sample and procedure

A cross-sectional study was carried-out two weeks after the beginning of Israel-Gaza war on the 7th of October 2023. Inclusion criteria were being an adult (aged over 18 years) from the general population of one of the five Arab countries involved in the study (i.e., Egypt, Jordan, Kuwait, Oman or Tunisia), capable of understanding the consent form and consenting to participate. An anonymous online survey and a snowball sampling method were adopted to collect data. The survey questionnaire was in the Arabic language, distributed to potential participants though social media platforms. It included in its first section general instructions, information about the research, and an informed consent form. The study protocol was approved by the ethics committee of Razi Psychiatric Hospital, Manouba, Tunisia, which is the home institution of the principal investigator [FFR] (Reference # ECRPH-2023-0068). The study was performed according to the Strengthening the Reporting of Observational Studies in Epidemiology (STROBE) guidelines [49].

### Minimal sample size

A minimal sample of 411 was deemed necessary using the formula suggested by Fritz and MacKinnon [50] to estimate the sample size: $=\frac{L}{f2}+k+1$, where f=0.14 for small effect size, L=7.85 for an α error of 5% and power β = 80%, and k=9 variables to be entered in the model.

#### Measures

The first part of the questionnaire contained sociodemographic information (sex, age, country of origin, marital status, educational level, and personal psychiatric history. The household crowding index was computed to reflect the socioeconomic status (i.e. the total number of individuals who live in the household divided by rooms’ number, excluding kitchens and bathrooms) [51]. The degree of financial satisfaction was also assessed on a 10-point Likert-type scale ranging from 1 (Not at all Satisfied) to 10 (very satisfied). The second part of the questionnaire was composed of the following measurement instruments:

##### The war-related media exposure scale (WarMES)

This is a newly developed scale by Fekih-Romdhane et al. [52]; it was designed and validated in the Arabic language to measure the intensity of war-related media exposure. Respondents were asked to indicate how frequently on average per day, over the past two weeks, they spend seeing each of the types of war-related content (e.g., “Victims under rubble”, “Families forcibly displaced from their homes”) on TV, radio, newspapers, magazines, or the Internet (e.g., television shows, breaking news, music videos, YouTube, Instagram, Facebook, TikTok). It is composed of 9 items scored on Five-point scale ranging from O (None) to 4 (More than 5 h per day). The scale yielded excellent psychometric properties (Cronbach’s alpha = 0.96).

##### The cohen perceived stress scale (PSS-10)

This is a 9-item self-report scale used to measure the degree to which respondents felt that life situations were stressful, overwhelming, uncontrollable, and unpredictable during the past month. Items are scored on a 5-point response scale ranging from 0 (never) to 4 (very often). Greater scores reflect higher levels of perceived stress. The Arabic validated version of the PSS-10 was used [53], with a Cronbach alpha of 0.678.

##### The patient health questionnaire–9 (PHQ-9)

This scale is composed of 9 items assessing and grading the severity of depression in the last 2 weeks [54]. The scale covers the 9 diagnostic criteria of the clinical diagnosis of depressive disorder according to the Diagnostic and Statistical Manual of Mental Disorders [54]. Each item is rated on a Likert scale from 0 (absence of symptom) to 3 (presence of symptom nearly every day). Higher scores indicate more severe depression. The Arabic version was used [55], which yielded a Cronbach alpha of 0.879 in the present sample.

**The insomnia severity index (ISI)**.

This scale is self-administered, and evaluates the nature, intensity and effects of insomnia through the following seven items: sleep maintenance, sleep dissatisfaction, severity of sleep onset, distress caused by the sleep difficulties, interference of sleep difficulties with daytime functioning, early morning awakening problems, and noticeability of sleep problems by others [56]. Greater scores indicate more severe insomnia. The Arabic validated version of the ISI was adopted [57], which exhibited a Cronbach alpha value of 0.792 for total scores.

### Statistical analysis

The SPSS software v.25 was used for the statistical analysis. The insomnia score was considered normally distributed since the skewness and kurtosis varied between − 1 and + 1; therefore, the score was dichotomized into absence and presence of suicidal ideation. The Student’s t-test was used to compare two means, whereas the Pearson test was used to correlate two continuous variables. The mediation analysis was conducted using PROCESS MACRO (an SPSS add-on) v.3.4 model 4; four pathways derived from this analysis: pathway A from the independent variable to the mediator, pathway B from the mediator to the dependent variable, Pathway C’ indicating the direct effect from the independent to the dependent variable. The results of the mediation analysis were adjusted over all variables that showed a *p* < .25 in the bivariate analysis. We considered the mediation analysis to be significant if the Boot Confidence Interval did not pass by zero. *P* < .05 was deemed statistically significant.

## Results

### Sociodemographic and other characteristics of the sample

A sample of 2635 participated in this study, with a mean age of 23.98 ± 7.55 years and 73.1% females. Other descriptive statistics of the sample can be found in Table 1.

**Table 1:** Sociodemographic and other characteristics of the sample (*N* = 2635)

Variable	*N* (%)
Country	
Jordan	349 (14.4%)
Egypt	910 (37.5%)
Tunisia	446 (18.4%)
Kuwait	571 (23.6%)
Oman	148 (6.1%)
Gender	
Male	709 (26.9%)
Female	1926 (73.1%)
Marital status	
Single	2093 (86.3%)
Married	331 (13.7%)
Education	
Secondary or less	145 (6.0%)
University	2279 (94.0%)
Personal history of psychiatric illness	
No	2135 (88.1%)
Yes	289 (11.9%)
	**Mean ± SD**
Age (years)	23.98 ± 7.55
Household crowding index (persons/room)	1.44 ± 1.00
Self-perceived financial burden	3.77 ± 2.75

### Bivariate analysis of factors associated with insomnia

The results of the bivariate analysis of factors associated with insomnia are summarized in Tables 2 and 3. The results showed that a higher mean insomnia score was found in participants who had a personal history of psychiatric illness vs. not. Moreover, older age was significantly associated with lower insomnia, whereas higher household crowing index, self-perceived financial burden, war media exposure, depression and perceived stress were significantly associated with more insomnia.

**Table 2:** Bivariate analysis of factors associated with insomnia

Variable	Mean ± SD	t	df	*p*
Gender		1.15	2633	0.250
Male	11.72 ± 5.85			
Female	11.43 ± 5.70			
Marital status		1.35	2578	0.177
Single	11.56 ± 5.72			
Married	11.17 ± 5.76			
Education		1.41	2633	0.157
Secondary or less	12.04 ± 5.38			
University	11.46 ± 5.78			
Personal history of psychiatric illness		-7.76	2633	**< 0.001**
No	11.17 ± 5.59			
Yes	14.12 ± 6.25			

Numbers in bold indicate significant *p* values

**Table 3:** Correlation matrix of continuous variables

	1	2	3	4	5	6	7
1. Insomnia	1						
2. Age	− 0.07***	1					
3. Household crowding index	0.04*	− 0.05*	1				
4. Self-perceived financial burden	0.04*	0.10***	0.02	1			
5. War media exposure	0.07***	0.05**	− 0.01	0.10***	1		
6. Depression	0.48***	− 0.10***	0.06**	0.08***	0.20***	1	
7. Perceived stress	0.33***	− 0.11***	0.06**	0.08***	0.13***	0.53***	1

*p < .05; **p < .01; ***p < .001

### Mediation analysis

**Fig. 1:**
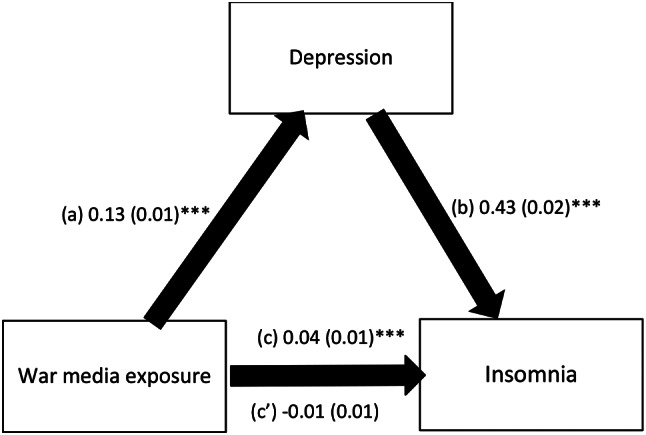
(a) Relation between war media exposure and depression (R^2^ = .095); (b) Relation between depression and insomnia (R^2^ = .239); (c) Total effect of war media exposure on insomnia (R^2^ = .040); (c’) Direct effect of war media exposure on insomnia. Numbers are displayed as regression coefficients (standard error). ***p < .001

**Fig. 2:**
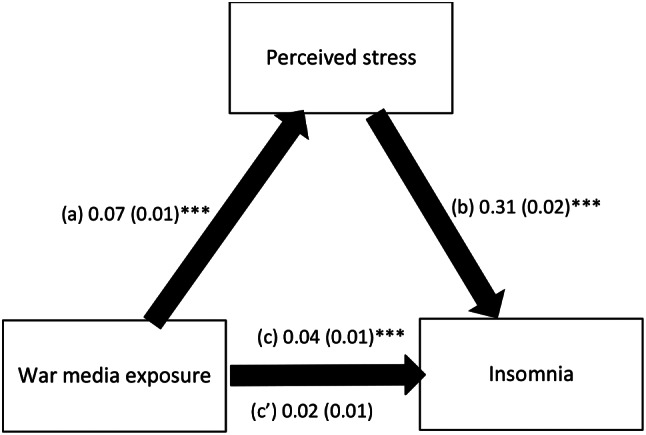
(a) Relation between war media exposure and perceived stress (R^2^ = .073); (b) Relation between perceived stress and insomnia (R^2^ = .127); (c) Total effect of war media exposure on insomnia (R^2^ = .040); (c’) Direct effect of war media exposure on insomnia. Numbers are displayed as regression coefficients (standard error). ***p < .001

The results of the mediation analysis are summarized in Table 4. The analysis was adjusted over the following variables: age, education, marital status, household crowding index, personal history of psychiatric illness and self-perceived financial burden. Depression and perceived stress fully mediated the association between war media exposure and insomnia; higher war media exposure was significantly associated with higher depression (Beta = 0.13; *p* < .001) and perceived stress (Beta = 0.07; *p* < .001), whereas higher depression (Beta = 0.43; *p* < .001) and perceived stress (Beta = 0.31; *p* < .001) were significantly associated with higher insomnia. It is of note that war media exposure was not significantly and directly associated with insomnia (Beta = − 0.01; *p* = .178 and Beta = 0.02; *p* = .098 respectively) (Figs. 1 and 2).

**Table 4:** Mediation analysis results, taking war media exposure as the independent variable, depression/perceived stress as the mediators and the insomnia as the dependent variable

	Direct effect			Indirect effect		
Mediator	Beta	SE	*p*	Beta	Boot SE	Boot CI
Depression	− 0.01	0.01	0.178	0.05	0.01	0.04; 0.07*
Perceived stress	0.02	0.01	0.098	0.02	0.004	0.016; 0.031*

*indicates significant mediation. Direct effect refers to the direct association between war media exposure and insomnia without the effect of the mediator, whereas the indirect effect refers to the same association through the mediators (depression / perceived stress)

## Discussion

Exposure to traumatic events, either directly [2], or indirectly through media coverage [13], were observed to often precipitate sleep disturbances. As available evidence on the topic remains relatively fragmented, mainly focused on posttraumatic stress outcomes and targeting Western populations [23, 38], more research is warranted to clarify whether and how exposure to media coverage related to war may be related to insomnia in the general population of non-Western countries. To this end, this study proposes to contribute to the literature available on impact of disaster media coverage on viewers by investigating, for the first time, the extent to which (1) media consumption is related to insomnia, and (2) depression and acute stress could (indirectly) affect this relationship among community adults of Arab origin in the immediate aftermath of the October 2023 Israel-Gaza war. Our hypothesis was supported, as analyses showed that both depression and acute stress served as mediators in the association between war media contact and insomnia. This suggests that media consumers of war content may be more at risk of insomnia when they experience more severe depression and stress.

This study is amongst the few to empirically investigate the relationship between war-related media exposure (across multiple media platforms) and insomnia in the immediate aftermath of a manmade disaster, which highlights its ecological validity and its potential to improve understanding of how early consumption of traumatizing media content may increase vulnerability to insomnia. In bivariate analyses, heavier media exposure to war content (in terms of hours per day) was positively associated with insomnia levels. One important strength of our study is the scale used for the assessment of media exposure, which is based on numeric quantification (time spent consuming coverage), as prior evidence suggested that using subjective (e.g., never, rarely, sometimes, often) or binary (e.g., yes, no) measurement scales may lead to underestimation of the association between media contact and mental health outcomes (e.g., [23]). Our findings align with the limited existing disaster- and non-disaster-related research in this area. Previous studies have shown that contact with violent media content was found to be significantly linked to sleep disturbances [10, 11]. Similar and closer to the present results, two prior studies found that found that early exposure to media coverage related to the 2011 Japanese earth-quake and tsunami [12] and to the 2015 Paris terrorist attacks [13] (two weeks and one month after their occurrence, respectively) were significantly associated with sleep disruption and insomnia. The first study involved participants from countries geographically far from the affected city, whereas the second study was performed among the general population of the country where the event occurred. The current study extends these observations to a different Non-Western Arab context and population, which have been largely underrepresented in the literature on this topic. Besides, Arab populations surveyed in this study are ethnically- and culturally-linked but geographically-distant to Palestinians, which provides new insights into media effects in such a new context. It is of note that, due to the cross-sectional design, the present findings should be interpreted with appropriate caution, and readers should bear in mind that future longitudinal studies are essential before establishing the temporal relationship between war media exposure and insomnia. Such research is especially needed given some evidence suggesting that the association between social media consumption and sleep disturbance could be bidirectional, with poor sleepers tending to use media as a sleep aid [58, 59].

Beyond exploring the bivariate association between war-related media exposure and insomnia, we sought to address the possible mediating mechanisms that may explain this relationship and pave the way for targeted interventions with increased efficacy. As anticipated, the two models of mediation were significant, with both depression and stress acting as significant mediators by which war media exposure is linked to insomnia symptoms. More particularly, participants with longer exposure time to media viewing of war reported more severe depression symptoms and exhibited stronger stress reactions; they tended, as a result, to have more severe insomnia symptoms. These findings, should they be confirmed by future longitudinal research, would suggest that a person’s insomnia level is influenced by the degree to which their psychological state has been affected by their contact with war media coverage experience. These findings are consistent with earlier research indicating that violent media exposure is associated with depression/stress symptoms [17, 23], and that the latter predict subsequent insomnia [24, 34]. It is of note that our analyses support total mediation, which signifies that depression and acute stress fully explain the association between media exposure and insomnia. However, it needs to be emphasized that bidirectional associations between stress, depression and insomnia were previously reported, both in non-disaster [60, 61] and disaster [62] research. Thus, conclusions can only be preliminary due to the cross-sectional design; future studies investigating alternative models with a longitudinal design are warranted to determine causal inference.

### Study limitations

This study has some limitations to be considered. Because of the cross-sectional nature, causation could not be established and prospective research is still required. Self-report measures were used for the assessment of sleep and not objective measures (e.g. actigraphy), which could have led to response bias. A selection bias is present for multiple reasons: (1) an online questionnaire and a snowball sampling technique were adopted, and might have resulted in limited representativeness of the sample to the wider general adult population, (2) the sample is not balanced between males and females, and (3) the number of participants is not randomized and does not represent all five Arab countries. Residual confounding bias is probable as well since not all factors associated with insomnia were taken into consideration in this study, therefore, results might be interpreted with caution. In addition, data related to the types of media and content themes were not collected. As participants consisted of adults of Arab origin and culture, the generalizability of findings to the broader general-population samples around the world still needs to be confirmed. Thus, future cross-cultural research needs to be conducted with media users from other countries, to examine whether individuals from multiple cultural contexts differ in their responses to exposure to war media content.

### Clinical and research implications

Since October 2023, the world population underwent massive exposure to immediate, unfiltered and disturbing war-related images from Gaza through all media sources. This unprecedented, constant exposure to vivid, unfiltered war content seems to have substantially affected large populations’ mental health further away from Gaza, and should therefore be considered a major global public health issue [63]. The present study is the first to provide evidence that, following this human catastrophe, more time spent viewing the horrors of war is significantly associated with insomnia. Sleep represents a fundamental human necessity for overall health, and a modifiable health behavior that is influenced by an array of individual (here, depression and stress) and environmental (here, war-related media exposure) factors. Identifying and targeting such factors through public health prevention efforts can help reduce insomnia in the general public during disaster times. Our results indicate that symptoms of stress and depression are present as early as two weeks following the beginning of the war, and play a role in mediating the association between war media coverage and insomnia. It is, therefore, suggested that the extent of exposure to war coverage should be assessed and monitored on a regular basis in people who experience insomnia in war times. Measures to decrease depression and stress in heavy media consumers may be potentially beneficial for preventing insomnia. In addition, once war coverage begins, the public should be informed that exposure may negatively affect mental health [64], and warned before war images are broadcasted [21].

## Conclusion

While our study leaves further research to be completed, it adds to the scant body of knowledge pertaining to the effect of media viewing of man-made disaster events on mental health​​. Analyses showed that depression and perceived stress act as full mediators in the relationship between war-related media exposure and insomnia. This suggests that timely screening for, and management of depression and stress symptoms in clinical and preventive programs might be beneficial for community adults who have been heavily and indirectly exposed to war through media, and present with insomnia. As wars and conflicts are not expected to subside in near future, communities and mental health professionals should be prepared to face the mental health crisis that would follow. Future prospective studies need to be conducted to confirm our findings and more accurately investigate causal influences of war media coverage on depression, stress and insomnia.

## Data Availability

The datasets generated and/or analyzed during the current study are not publicly available due to restrictions from the ethics committee but are available from the corresponding author on reasonable request.
